# Do Single Food Habits Matter? Fish and Vegetables Intake and Risk of Low HRQoL in Schoolchildren (ASOMAD Study)

**DOI:** 10.3390/children13010056

**Published:** 2025-12-30

**Authors:** Alicia Portals-Riomao, Asmaa Nehari, Marcela González-Gross, Carlos Quesada-González, Eva Gesteiro, Augusto G. Zapico

**Affiliations:** 1 ImFINE Research Group, Department of Health and Human Performance, Universidad Politécnica de Madrid, 28040 Madrid, Spainmarcela.gonzalez.gross@upm.es (M.G.-G.); carlos.quesada@upm.es (C.Q.-G.); eva.gesteiro@upm.es (E.G.); a.gzapico@upm.es (A.G.Z.); 2 Physical Exercise and Health Research Network, EXERNET, 18016 Madrid, Spain; 3 Biomedical Research Center of Pathophysiology of Obesity and Nutrition (CIBERobn), Carlos III Health Institute, 28029 Madrid, Spain; 4 Department of Mathematics Applied to Information and Communication Technologies, Universidad Politécnica de Madrid, 28031 Madrid, Spain

**Keywords:** children, health-related quality of life, vegetables, fish, Mediterranean diet, Generalised Estimating Equations, marginal probabilities, physical activity, screen time, socioeconomic status

## Abstract

**Highlights:**

**What are the main findings?**

• In Madrid children aged 8–12, eating vegetables ≥2/day and fish ≥2–3/week was associated with a lower risk of KIDSCREEN <40; adjusted probabilities: 40.1% (neither), 25.8% (vegetables only), 29.7% (fish only), 34.0% (both). The combined effect was smaller than the sum of the separate effects.
• Moderate-to-vigorous physical activity was protective; recreational screen time was detrimental.

**What are the implications of the main findings?**

• Two concrete, feasible targets for school canteens and households, alongside more physical activity and less screen time.
• Causal validation is needed, with an equity focus by SES and by school.

**Abstract:**

Background/Objectives: Evidence links children’s health-related quality of life (HRQoL) to overall diet, but data on specific, actionable habits are limited. We tested whether vegetable intake ≥2 portions/day and fish intake ≥2–3 times/week were associated with risk of low HRQoL (KIDSCREEN-10 Index score <40) and assessed their joint effect and robustness to overall diet quality. Methods: In three waves (2020–2023) in Madrid (Spain), 1127 observations from 771 children (8–12 years) were analysed. Logistic Generalised Estimating Equations (GEE) adjusted for age, sex, socioeconomic status (four levels), moderate-to-vigorous physical activity (MVPA), screen time, body mass index (BMI) z-score, wave and school ownership. Marginal predicted probabilities were computed for four exposure combinations (neither, vegetables only, fish only, both). Sensitivity models added school area and the Mediterranean Diet Quality Index (KIDMED; KIDMED_wo_FV and total); hybrid within–between GEE and a linear mixed model for continuous KIDSCREEN-10 were also fitted. Results: Vegetables ≥2/day and fish ≥2–3/week were inversely associated with low HRQoL (odds ratio (OR) 0.49 (95% confidence interval (CI) 0.30–0.82) and 0.61 (0.43–0.87)). The interaction was positive (OR 2.50 (1.39–4.53)). Adjusted probabilities were 40.1% (neither), 25.8% (vegetables only; −14.3 percentage points (p.p.)), 29.7% (fish only; −10.5 p.p.), and 34.0% (both; −6.1 p.p.). Findings persisted with KIDMED_wo_FV and attenuated with total KIDMED. MVPA related inversely and screen time directly to risk. Conclusions: Vegetables ≥2/day and fish ≥2–3/week were associated with lower odds of low HRQoL, with non-additive combined effects. These simple targets may complement physical-activity promotion and reduced screen time; longitudinal/experimental studies should test causality and dose–response.

## 1. Introduction

Child health-related quality of life (HRQoL) is a key determinant of current and future health, with implications for school performance and family dynamics, and it imposes a substantial burden on health systems and communities [1]. Previous observational studies in young people have linked diet quality and specific foods with internalising difficulties, such as depressive and anxiety symptoms [2]. In particular, higher fruit and vegetable intake has been associated with fewer depressive/anxiety symptoms, and fish intake has been examined in relation to depressive symptoms, although findings vary across settings and measures [3].

Physical activity shows consistent benefits, whereas higher screen time is prospectively associated with poorer perceived well-being/HRQoL and more depressive symptoms [4,5,6]. Regarding diet, evidence has focused mainly on overall dietary quality or adherence to patterns such as the Mediterranean diet, showing associations but heterogeneous findings in young people [5,7]. However, there is a shortage of studies on specific, actionable habits (e.g., fish or vegetable intake) that can support simple, comparable recommendations in school and community settings [8]. In the present study, HRQoL was operationalised using the KIDSCREEN-10 Index, a measure of general HRQoL, rather than a clinical symptom scale [9].

The focus is placed on fish and vegetables, rather than the total KIDMED score, for biological and practical reasons. Against this background, this study focused on two simple behaviours—fish intake ≥2–3 times/week and vegetable intake ≥2 portions/day—that can be communicated and monitored in school/community settings. Fish, particularly oily fish (e.g., sardines, anchovies, mackerel, salmon, trout and herring), is an important dietary source of long-chain omega-3 polyunsaturated fatty acids (EPA and DHA), which are implicated in neurodevelopment and emotion regulation. However, omega-3 content varies markedly by species and preparation, and many commonly consumed lean fish contain substantially lower amounts; therefore, our fish-frequency exposure should be interpreted as a pragmatic proxy for habitual fish intake rather than as a direct measure of omega-3 intake. Beyond omega-3, fish also provides high-quality protein and key micronutrients (e.g., iodine, selenium and vitamin B12), which may be relevant to child health [10,11,12]. In Spain and in the community of Madrid, household purchase/consumption reports indicate that commonly consumed fish includes a mix of lean and oily species (e.g., hake/whiting and salmon, as well as small pelagic fish such as sardines/anchovies), supporting the interpretation of our exposure as “overall fish intake” rather than “omega-3 intake" [13,14]. Vegetables provide a range of dietary fibres, including an insoluble fraction and a fermentable fraction (e.g., pectins, inulin-type fructans and resistant starch), alongside polyphenols (e.g., flavonoids and phenolic acids) and micronutrients such as folate, magnesium and vitamin C [15]. The fermentable fibre fraction is metabolised by the gut microbiota, increasing short-chain fatty acids (SCFAs; acetate, propionate and butyrate), which support intestinal barrier integrity and modulate immune and inflammatory pathways, with downstream relevance to gut–brain signalling [16,17]. Dietary polyphenols are also biotransformed by gut microbes into bioactive metabolites, and together these diet–microbiota products may influence oxidative stress and neuroinflammatory processes [18,19,20]. These are straightforward to communicate as public health messages (e.g., “≥2–3 times/week of fish”, “≥2 portions/day of vegetables”), consistent with public health guidance [21,22]. By contrast, KIDMED index reflects overall Mediterranean diet adherence and includes fish and vegetable items. Because fish and vegetables were our focal exposures, adjusting for total KIDMED could lead to over-adjustment and collinearity; therefore, we used KIDMED only in sensitivity analyses, including a version excluding those items [23,24,25].

The literature in children and adolescents reports associations between dietary quality and well-being/HRQoL, with a predominance of cross-sectional studies and some cohorts, and heterogeneous results [26,27,28]. Evidence is more limited when fish and vegetables are assessed as individual behaviours; for fruit/vegetables, reviews note a scarcity of interventions and a need for more robust designs, and for fish there are few specific studies with partial findings on behaviour and pro sociality [8,29]. Dietary exposures are commonly measured using food-frequency questionnaires (FFQ), 24 h recalls or records and indices such as KIDMED; in some cases, single items are used [30,31]. There is heterogeneity in ages, outcome definitions and confounder control, and reviews call for more prospective studies [26,28]. Gaps remain: (i) testing the combined fish × vegetable effect, (ii) demonstrating independence from overall diet quality and (iii) separating between-child differences from within-child changes over time (limited longitudinal evidence using a within–between approach) [8,28].

A multidomain framework is adopted in which diet relates to HRQoL alongside daily moderate-to-vigorous physical activity (MVPA)—typically protective—and screen use, which is prospectively and experimentally linked to poorer psychological health; contextual determinants such as adiposity and socioeconomic status (SES) shape both habit acquisition and risk expression [4,32,33,34,35]. From this perspective, fish and vegetable intakes should be interpreted within that behavioural and social framework, avoiding simplistic attributions and considering possible overlaps of effects across behaviours and social gradients [4,32,33]. It is also conceptually plausible that their combination is not strictly additive (e.g., sub-additivity), which justifies considering their joint effect in research [36].

The primary aim was to estimate, at the population level, the association between two achievable behaviours—fish intake (≥2–3 times/week) and vegetable intake (≥2 times/day)—and the risk of low HRQoL (KIDSCREEN <40), independently of other behaviours and socioeconomic context. Secondary aims were to assess the joint effect (fish × vegetable interaction) to distinguish synergy, additivity or sub-additivity and, longitudinally, to differentiate whether within-child variations and sustained intake show distinct patterns. Taken together, both behaviours are expected to be inversely associated with the risk of low HRQoL, with a combined benefit that is not strictly additive.

## 2. Materials and Methods

### 2.1. Study Design and Setting

A city-wide, school-based study was conducted in Madrid (Spain) among pupils aged 8–12 years. Data were collected across three academic waves (2020–2021, 2021–2022, 2022–2023), each during late autumn to winter. The design uses repeated observations for a subset of participants (one to three per child), noting the evolving public health context after COVID-19 while keeping measures comparable across waves. Procedures followed standardised field protocols and appropriate safety measures.

#### 2.1.1. Sampling, Participants and Recruitment

A stratified, multi-stage cluster sampling approach was applied to ensure city representativeness across socioeconomic strata and school ownership (public, charter/state-subsidised and private). Primary units were classrooms (about 25 pupils), with proportional allocation by municipal income zones and ownership distribution (approximately 40/40/20%). Eligibility required enrolment in participating schools and parental consent; severe learning disability was an exclusion criterion. Recruitment aimed for broad geographic coverage across Madrid districts and zones.

#### 2.1.2. Procedures and Fieldwork

Trained staff conducted on-site assessments during school hours using validated instruments and harmonised questionnaires completed by pupils and parents or guardians. Where relevant, parental questionnaires were adapted from nationally used tools to support comparability with prior Spanish cohorts. Daily quality checks were run to identify inconsistencies and ensure data completeness before closing the database.

### 2.2. Measures

#### 2.2.1. Primary Outcome: HRQoL

HRQoL was assessed using the KIDSCREEN-10 Index, scored as standardised T-values (norm mean fixed at 50, SD 10; higher values indicate better HRQoL). Low HRQoL was defined a priori as a KIDSCREEN-10 T-score < 40 [37], corresponding to approximately one standard deviation below the norm mean and commonly interpreted as low HRQoL in population-based studies. The Spanish version of the KIDSCREEN-10 has shown adequate psychometric properties supporting its use in Spanish youth samples [9]. Wave-specific z-score checks were used as standardisation diagnostics and not as outcomes [38,39].

#### 2.2.2. Dietary Exposures of Interest

Two actionable behaviours were analysed as stand-alone exposures derived from two brief frequency items (screening proxies rather than a quantitative dietary assessment): (1) fish intake of at least two to three times per week (versus fewer), and (2) vegetable intake of at least two portions per day (versus fewer). These items align with Mediterranean diet screening constructs used in the KIDMED questionnaire (developed in Spain), which includes items on fish consumption ≥2–3 times/week and vegetables more than once/day [40]. Although we analysed these behaviours independently (i.e., not as part of a composite diet score), they should be interpreted as pragmatic proxies for habitual intake and may be subject to misclassification; any non-differential misclassification would be expected to attenuate associations towards the null. In support of their use in Spanish settings as low-burden screening indicators, psychometric evaluation of the updated KIDMED instrument in Spanish youth has reported acceptable test–retest reliability and item-level agreement against a 7-day dietary record, with variability across items [25].

The KIDMED index was computed and used only in sensitivity analyses—including a version excluding fish and vegetable items—to minimise over-adjustment and multicollinearity when fish and vegetables were the focal exposures [25,40]. To adjust for overall diet quality without conditioning on the exposures, we created a modified KIDMED score excluding the fish and vegetable items: “KIDMED_wo_FV” = KIDMED − fish − veg2x (with fish and veg2x coded 1 if the threshold is met and 0 otherwise); all other KIDMED items remained unchanged.

#### 2.2.3. Other Lifestyle Variables

Physical activity was assessed with PAU-7S and expressed as moderate-to-vigorous physical activity (MVPA) in minutes per day; a derived indicator of meeting the 60 min/day guideline was used descriptively [41]. Screen time was measured with the SSBQ, recording daily minutes on TV, computers, consoles and smartphones, separately for weekdays and weekends; a minutes-per-day metric was used in models [42,43,44].

#### 2.2.4. Anthropometry and Adiposity

Height and waist were measured with SECA devices; weight and percentage body fat came from bioimpedance (Tanita DC-240MA). Body mass index (BMI) was computed as kg/m^2^ and age-standardised using international references [45]. Waist-to-height ratio (WHtR) used the 0.5 cut-off to indicate central adiposity [46]. Where appropriate, z-BMI (BMI z score) was entered as a continuous covariate.

#### 2.2.5. Sociodemographic and Contextual Variables

Models included age (months), sex, school ownership (public/charter/private), wave (1–3), and socioeconomic status (SES). A school-zone variable (low/medium/high area income) was used in sensitivity analyses to capture contextual gradients beyond individual SES [47,48,49].

#### 2.2.6. Socioeconomic Status (SES) Index

A multidimensional SES index combined parental education, occupational class and area income. Education was mapped to ISCED and grouped as low, medium or high. Occupation was mapped to ISCO and grouped as low, medium–low, medium–high or high following common practice in paediatric epidemiology [50,51]. For area income, school-census section data for the corresponding year were used to assign regional quintiles (1–5) [52]. Dimension scores were summed into a composite range (3–12) and then grouped into four ordered categories: G1 = 3–5 (low), G2 = 6–7 (medium–low), G3 = 8–9 (medium–high), G4 = 10–12 (high). This simple grouping supports stable contrasts [53].

#### 2.2.7. Sample Size and Precision

Sampling was planned around classroom clusters (about 25 pupils), a 70% expected participation rate, balanced representation across income zones and school ownership. This gave a target of roughly 360 pupils per wave, with extra recruitment to account for attrition. Given the mixed cross-sectional and short-panel structure, repeated observations per child were expected (one to three). Precision for adjusted associations was evaluated after analysis via 95% confidence intervals in the modelling framework below.

### 2.3. Statistical Analysis

#### 2.3.1. Primary Model

Primary analyses were conducted using complete records for variables included in each model; missing-data handling and analytic sample derivation are described in Section 2.3.6. To estimate population-averaged associations with low HRQoL (KIDSCREEN < 40), we fitted a logistic Generalised Estimating Equations (GEE) model (logit link) with an exchangeable working correlation and robust (sandwich) standard errors clustered by child ID. The model included main effects for vegetables ≥2/day and fish ≥2–3/week plus their interaction (vegetables×fish) and was adjusted a priori for age (months), sex, SES (four levels), MVPA (min/day), screen time (min/day), BMI z-score, wave (1–3) and school ownership. Results are presented as odds ratios (OR) with 95% CIs and two-sided *p*-values.

An exchangeable working correlation was selected as a parsimonious and stable choice because the number of repeated observations per child was small and unbalanced (most children contributed one observation). As a robustness check, we refitted the primary model using alternative working correlation structures (independent and AR(1), where estimable); key exposure estimates were materially unchanged (Supplementary Table S5). An unstructured correlation was not pursued because the small cluster sizes (predominantly 1–2 observations per child) may yield unstable estimates in practice.

#### 2.3.2. Marginal Predictions

From the primary GEE, marginal predicted probabilities were obtained for the four exposure scenarios—00 (neither), 10 (vegetables only), 01 (fish only), 11 (both)—using marginal standardisation (covariates kept at their observed distributions). Risk differences and relative differences were computed with 95% CIs.

#### 2.3.3. Within–Between (Hybrid) Analysis

To distinguish within-child changes from between-child differences, each exposure was decomposed into the child-specific mean (between component) and the deviation from that mean at each observation (within component), following a Mundlak-type hybrid specification within the same GEE framework.

#### 2.3.4. Sensitivity and Threshold Analyses

Pre-specified sensitivity analyses included: (i) additional adjustment for school zone (low/medium/high); (ii) inclusion of KIDMED_wo_FV (per-point) to account for overall diet quality without conditioning on the fish/vegetable exposure items; and, as supplementary checks, models additionally adjusted for the total KIDMED score and models using KIDMED tertiles; (iii) alternative exposure thresholds (e.g., vegetables ≥ 1/day, fish ≥ 1/week) to explore potential dose–response patterns. Because fish and vegetables are items within KIDMED, results from models adjusting for total KIDMED are interpreted as conservative and are presented to illustrate the expected attenuation due to construct overlap (Supplementary Figures S4 and S5).

#### 2.3.5. Continuous Outcome

For the continuous KIDSCREEN score, a linear mixed-effects model with a random intercept for child was fitted using the same adjustment set.

#### 2.3.6. Missing Data

Missing data were handled as follows. Among observations with non-missing outcome and exposures (N = 1127), missingness in continuous covariates used in the fully adjusted model was negligible (≤0.1% for age, screen time and BMI z-score), yielding an analytic sample of N = 1125 (Supplementary Table S6). Socioeconomic status (SES) had 11.4% missingness and was retained by including an explicit ‘Missing’ category (Table 1). As a robustness check, we refitted the primary model excluding observations with missing SES (N = 999) and obtained materially similar estimates for vegetables, fish, and their interaction (Supplementary Table S7). Multiple imputation was not pursued because missingness was minimal for most covariates and results were robust to the SES-missing sensitivity analysis; nonetheless, some bias remains possible if SES non-response is related to both exposure and outcome.

#### 2.3.7. Software and Reproducibility

Analyses were conducted in Python (version 3.9) 3.12 using pandas (data handling), NumPy (numerics), statsmodels 0.14 (GEE, MixedLM, marginal effects) and Matplotlib (figures). Data cleaning recoded sentinel values (−99), created derived variables (5:2 weekday–weekend means; SES grouping; binary exposures; KIDMED_wo_FV; within/between decompositions) and restricted models to complete cases. Reporting follows STROBE guidelines.

## 3. Results

A total of 1127 observations from 771 children were available (1–3 per child; mean cluster size 1.46). In the analytical sample, the prevalence of the exposures at observation level was 40.6% for vegetables ≥2/day and 71.6% for fish ≥2–3/week; the prevalence of low HRQoL (KIDSCREEN < 40) was 31.7% (Table 1).

For the fully adjusted regression models, the analytic sample was 1125 observations due to minimal covariate missingness (Supplementary Table S6); findings were robust when excluding observations with missing SES (Supplementary Table S7).

**Figure 1:**
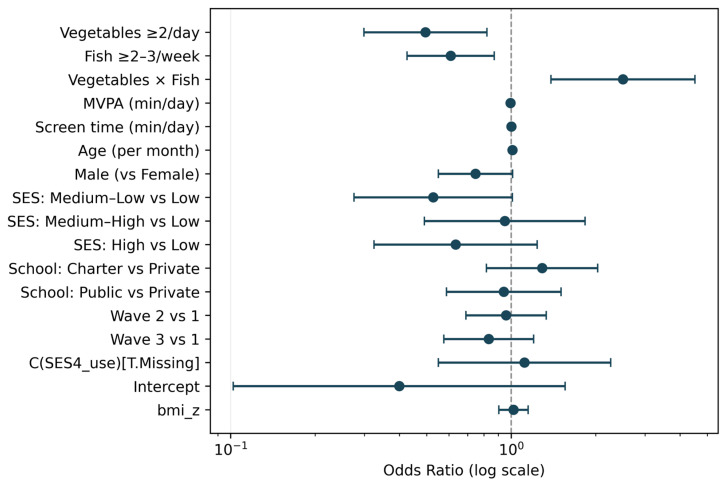
Forest plot—Primary GEE. Notes. Logistic GEE with exchangeable working correlation and robust (sandwich) standard errors clustered by child ID. Adjusted for age (months), sex, socio-economic status (four levels), MVPA (min/day), screen time (min/day), BMI z-score, wave (1–3) and school ownership. The x-axis is on a log scale; values < 1 indicate lower odds.

Primary model (population-averaged GEE with robust SEs clustered by child ID.). After adjusting for age (months), sex, socio-economic status (four levels), MVPA (min/day), screen time (min/day), BMI z-score, wave and school ownership, both behaviours were inversely associated with low HRQoL: vegetables ≥ 2/day, OR 0.49 (95% CI 0.30–0.82; *p* = 0.006); fish ≥ 2–3/week, OR 0.61 (0.43–0.87; *p* = 0.006) (Figure 1). The vegetables × fish interaction term exceeded 1, indicating sub-additivity on the odds scale; the combined pattern was associated with a smaller-than-expected difference (interaction OR 2.50; 1.39–4.53; *p* = 0.002–0.003). Among covariates, age per month: OR 1.011 (*p* = 0.036); MVPA per 60 min: OR 0.71 (*p* < 0.001); screen time per 60 min: OR 1.09 (*p* = 0.004). The coefficient for boys vs. girls was borderline (OR 0.74; *p* = 0.053). SES categories (vs G1), wave, school ownership and BMI z-score were not statistically significant.

**Figure 2:**
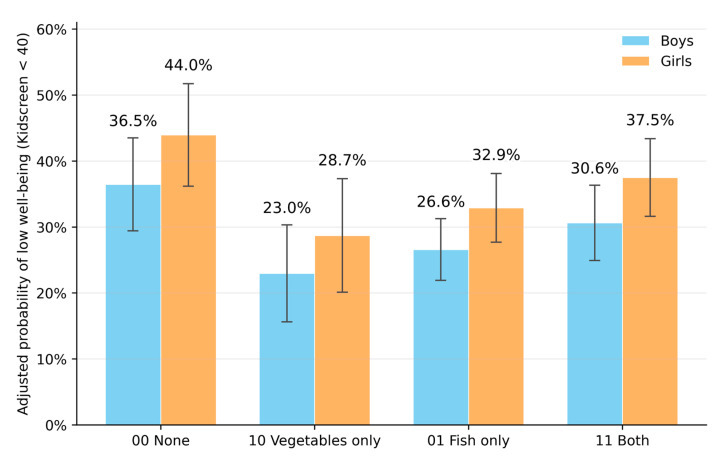
Adjusted probability of low HRQoL (KIDSCREEN < 40) by sex across exposure combinations. Notes . Marginal predicted probabilities from the primary GEE model using marginal standardisation. Scenarios: 00 = neither behaviour, 10 = vegetables ≥2/day only, 01 = fish ≥2–3/week only, 11 = both behaviours. Bars show point estimates; whiskers show 95% confidence intervals.

Adjusted marginal probabilities (combined scenarios). Estimated probabilities for KIDSCREEN < 40 were: 00 (none) 40.1% (95% CI 33.4–46.8), 10 (vegetables only) 25.8% (18.2–33.3), 01 (fish only) 29.7% (25.6–33.8) and 11 (both) 34.0% (29.0–39.0). These margins illustrate a less-than-additive combined effect: although the estimated risk was lower in ‘both’ than ‘neither’, the difference was smaller than expected from adding the separate effects, consistent with antagonism on the odds scale. Absolute differences vs. 00 were −14.3 percentage points for 10 (95% CI −24.2 to −4.5), −10.5 for 01 (−18.2 to −2.8) and −6.1 for 11 (−14.5 to +2.2). Pattern by sex is shown in Figure 2; overall margins are given in Supplementary Figure S1.

Stratified marginal probabilities (Supplementary Materials). The ordering 10 < 01 < 11 < 00 was observed by sex, SES, wave and school ownership. For girls: 44.0% (00), 28.7% (10), 32.9% (01), 37.5% (11). For boys: 36.5%, 23.0%, 26.6%, 30.6%. For SES, examples: G1 55.0% (00) vs. G4 33.3% (00). Across waves, 00 was 43.0%, 40.2% and 38.0% in waves 1–3, respectively. For school ownership, 00 was 32.5% (private), 43.1% (charter) and 41.9% (public). Stratified probabilities by SES are shown in Supplementary Figure S2, and detailed estimates by sex, SES group, wave and school ownership are provided in Supplementary Tables S1–S4.

Within–between (hybrid GEE, Mundlak). Within-child deviations were not associated with the outcome: vegetables within OR 0.95 (0.64–1.41; *p* = 0.796); fish within OR 1.23 (0.74–2.05; *p* = 0.417). Between-child means were fish mean OR 0.72 (0.51–1.02; *p* = 0.064) and vegetables mean OR 0.93 (0.66–1.30; *p* = 0.670). MVPA remained inversely associated (*p* = 9.46 × 10^−5^) and screen time directly associated (*p* = 0.0058). Forest plots for these models are available in Supplementary Figures S3–S5.

Continuous outcome (linear mixed model for KIDSCREEN score; random intercept for child. Coefficients for the exposures were not significant: vegetables ≥2/day β 0.149 (95% CI −0.464 to 0.762); fish ≥2–3/week β 0.656 (−0.037 to 1.349). Significant covariates were age β −0.037 (−0.061 to −0.014), MVPA β 0.0138 (0.0080–0.0196) and screen time β −0.0056 (−0.0082 to −0.0030).

Sensitivity analyses. (a) Additional adjustment for school area (low/middle/high): vegetables OR 0.50 (0.30–0.83; *p* = 0.0076); fish OR 0.61 (0.43–0.88; *p* = 0.0076); interaction OR 2.48 (1.37–4.49; *p* = 0.0027); age OR 1.012 (*p* = 0.027); MVPA (*p* = 7.67 × 10^−5^) and screen time (*p* = 0.0068) remained associated. (b) Including KIDMED without the fish/vegetable items (per point): vegetables OR 0.54 (0.32–0.89; *p* = 0.0148); fish OR 0.66 (0.46–0.95; *p* = 0.0273); interaction OR 2.42 (1.35–4.36; *p* = 0.0032); KIDMED_wo_FV OR 0.889 (0.832–0.950; *p* = 0.00055). (c) Including total KIDMED: vegetables OR 0.60 (0.36–1.00; *p* = 0.0516); fish OR 0.75 (0.51–1.09; *p* = 0.132); interaction OR 2.42 (1.35–4.36; *p* = 0.0032); KIDMED total OR 0.889 (0.832–0.950; *p* = 0.00055). (d) Alternative exposure (KIDMED tertiles): vs. lowest tertile, middle OR 0.66 (0.49–0.88; *p* = 0.0056) and highest OR 0.50 (0.34–0.75; *p* = 0.00072). In this model, boys vs. girls OR 0.70 (0.52–0.95; *p* = 0.024); MVPA (*p* = 0.00031) and screen time (*p* = 0.024) were associated.

## 4. Discussion

We examined whether two achievable dietary behaviours—vegetables ≥2/day and fish ≥2–3/week—were associated with the population-level risk of low perceived well-being/HRQoL (KIDSCREEN < 40). Both showed inverse associations after multivariable adjustment (vegetables OR 0.49; fish OR 0.61), with lower estimated absolute risks reductions versus neither behaviour (−14.3 and −10.5 percentage points, respectively). The vegetables×fish interaction was positive (OR ≈ 2.50), indicating sub-additivity whereby combined adherence does not fully add benefits. Results were robust across sensitivity analyses, highlighting pragmatic, attainable targets with potential public health relevance for children’s well-being.

Several complementary pathways may link fish and vegetable intake with health-related quality of life (HRQoL) in school-aged children. Fish intake may relate to neurodevelopmental and immunomodulatory processes that remain active throughout mid-childhood, including neuronal membrane function, synaptic plasticity and inflammatory signalling, while acknowledging that nutrient profiles vary by fish species and preparation [54]. Vegetables may contribute to HRQoL partly via the gut–brain axis: fermentable fibres and polyphenols can modulate the gut microbiota and microbial metabolites (e.g., short-chain fatty acids), supporting intestinal barrier function and influencing immune and oxidative stress pathways, which may be relevant to HRQoL [55]. Behaviourally, higher intake of fish and vegetables may also displace ultra-processed, energy-dense foods and added sugars; dietary patterns characterised by higher ultra-processed food intake have been linked to higher levels of inflammatory biomarkers in children/adolescents, and umbrella evidence also links ultra-processed food exposure to a range of adverse health outcomes [56,57]. The positive vegetables×fish interaction (sub-additivity on the odds scale) may reflect partially overlapping pathways and diminishing returns when one pathway is already engaged; however, interpretation of interaction depends on the effect scale and should be treated as suggestive rather than mechanistic proof [58]. The independent associations observed for MVPA (protective) and screen time (risk) align with a multicomponent model of child HRQoL, in which diet, movement behaviours and sedentary exposure jointly contribute to perceived well-being/HRQoL [43,59,60].

The positive vegetables×fish interaction indicates sub-additivity on the odds scale, meaning that the combined association is smaller than would be expected from multiplying the separate odds ratios. Importantly, interaction depends on the effect scale and should not be interpreted as mechanistic antagonism; a departure from multiplicativity on the odds scale may coexist with additivity on an absolute risk scale [36]. One plausible explanation is diminishing returns due to partially overlapping pathways: fish intake may relate to neurodevelopmental and immunomodulatory processes, while vegetables may act partly through the gut–brain axis via fermentable fibres and polyphenols that influence microbial metabolites and inflammatory/oxidative stress signalling [18,54]. Sub-additivity could also reflect heterogeneity in the ‘fish’ exposure (lean vs. oily species) and non-differential misclassification from brief frequency items, which can attenuate main effects and distort interaction estimates [25,31]. Finally, residual confounding by broader family food environments and correlated behaviours may contribute, such that the ‘both’ group is not simply the sum of two independent exposures. Future work with more granular dietary measures (species/portion size), repeated exposure assessment and biomarkers of inflammation could test whether this pattern reflects pathway overlap or measurement/selection artefact [56,57].

International evidence is consistent with these associations. In Greek adolescents, greater adherence to Mediterranean diet habits was positively related to HRQoL assessed with KIDSCREEN-27 [61]. In Portuguese adolescents, higher Mediterranean diet adherence was associated with higher HRQoL measured with KIDSCREEN-10 (B = 0.259, 95% CI 0.140–0.377) [62]. A systematic review of observational studies in children and adolescents also concluded that Mediterranean diet adherence is generally positively associated with HRQoL, although effect sizes and measures are heterogeneous [63].

Quantitatively, in a 1371-child prospective cohort from Catalonia, each 1-point higher KIDMED score at baseline was associated with a 0.32-point higher KIDSCREEN-10 at 15 months (β = 0.320; 95% CI 0.101–0.540), and baseline fruit/vegetable consumption independently predicted higher HRQoL at follow-up [64]. Crucially, in our sensitivity model that accounted for overall diet quality excluding the fish/vegetable items, associations for vegetables (OR 0.54; 95% CI 0.32–0.89) and fish (OR 0.66; 0.46–0.95) persisted, while the KIDMED_wo_FV score showed an independent protective association (per-point OR 0.889; 0.832–0.950), indicating food-specific pathways beyond the global pattern.

At the same time, trials of long-chain n-3 supplementation in young people with depressive symptoms show mixed and often small effects, with recent syntheses rating the certainty as low to very low—compatible with our population-level observational associations coexisting with modest average treatment effects at the individual level. Observational studies also link higher fruit/vegetable intake with better HRQoL scores in school settings, consistent with our vegetable finding and the notion that combinations of nutrient-dense foods (rather than scores alone) matter for HRQoL [11,65].

Despite significant associations in the binary models, the linear mixed model with continuous KIDSCREEN did not detect effects of fish or vegetables. A plausible explanation is non-linearity with a threshold around one SD below the norm (T ≈ 40), where risk differences concentrate; modelling the full scale can dilute such changes. Additional factors include loss of precision from the scale’s restricted range, subgroup heterogeneity and dietary measurement error. Going forward, we recommend modelling potential curvatures using restricted cubic splines or fractional polynomials, and analysing KIDSCREEN as an ordinal outcome (proportional-odds), which preserves ordering and may improve power to detect distributional shifts [38,66,67].

GEE provides average across-children estimates; our hybrid within–between (Mundlak) model indicated no short-term within-child effect of fish/vegetable intake, suggesting that associations are driven largely between children, consistent with relatively stable dietary patterns and unmeasured family-level factors. Causal interpretation should therefore be cautious given potential residual confounding (e.g., home emotional climate, parenting, food availability), limited temporal variability in exposure (mean 1.46 observations/child) and non-differential dietary misclassification from brief frequency items, which typically bias effects towards the null [68]. Although SES, wave and school ownership were not statistically significant after adjustment, our stratified margins showed differing baselines (e.g., higher 00 risk in G1), implying that context still shapes absolute risk even when relative associations appear similar [69]. We found no evidence of effect modification by sex, SES or school ownership (all *p* for interaction > 0.10), although baseline absolute risks differed across strata, as shown in stratified margins (Supplementary Tables S1–S4).

Strengths include a sizeable urban cohort with repeated observations across three waves (2020–2023); comprehensive adjustment for confounders (MVPA, screen time, BMI z-score, SES, age, sex, wave and school ownership); multiple sensitivity analyses (additional school-area adjustment; KIDMED models excluding fish/vegetable items and using total score; KIDMED tertiles as alternative exposure); and estimation of absolute effects via marginal standardisation. Limitations include the observational design and dietary exposures measured using two brief frequency items rather than a full quantitative dietary assessment. As such, they serve as proxies for habitual intake and cannot capture portion size (grams), preparation methods or overall dietary pattern with precision. Any resulting non-differential misclassification would be expected to attenuate associations towards the null, so effect sizes may be conservative [70]. Limitations also include limited within-child repetitions (mean 1.46/child), potential selection bias and concurrent measurement of exposures and outcome, which together temper causal claims. Practically, ~10–14 percentage-point lower risk relative to neither behaviour frames ≥2/day vegetables and ≥2–3/week fish as feasible, low-cost targets within multicomponent approaches (diet + more MVPA + less screen time). School actions could prioritise canteen menus with weekly fish offerings, prominent vegetable availability and food education; families can support through menu planning, shopping lists and simple, budget-aware recipes. An equity lens is essential: tailor supports by SES and school context (e.g., affordable procurement, subsidies, culturally adapted recipes, default healthy options) so gains are shared across private, charter and public settings.

Despite significant associations in the binary models, the linear mixed model using continuous KIDSCREEN-10 did not provide clear statistical evidence for associations with fish or vegetables. One possible explanation is that any relationship is non-linear and concentrated in the lower tail of the distribution (around ~1 SD below the norm mean; T ≈ 40), such that modelling the full scale may dilute changes relevant for identifying markedly low HRQoL. Additional considerations include limited within-child variability, subgroup heterogeneity, restricted range of the outcome and dietary measurement error. Future work could test potential non-linearity using restricted cubic splines or fractional polynomials and consider ordinal models that preserve ranking across the distribution.

## 5. Conclusions

Vegetable intake ≥2 portions/day and fish intake ≥2–3 times/week were each independently associated with lower odds of low HRQoL (KIDSCREEN < 40), with adjusted ORs of 0.49 and 0.61 and lower estimated risk (absolute risk differences) of roughly 14 and 11 percentage points versus neither behaviour. A positive vegetables×fish interaction indicated sub-additivity, and results were robust to multiple sensitivity analyses, including adjustment for overall diet quality using a KIDMED variant excluding the fish/vegetable items. Hybrid within–between models suggested that associations reflect between-child differences rather than short-term within-child changes; higher MVPA and lower screen time were consistently related to better HRQoL. Overall, these findings suggest that these behaviours may be useful pragmatic targets and could be considered within multicomponent school and family programmes, while highlighting the need for longitudinal and experimental studies with finer dietary measurement to test causality and dose–response. Given the observational design and short, unbalanced panel, these findings should be interpreted as population-averaged associations and do not support causal inference.

## Figures and Tables

**Table 1:** Sample characteristics (observation-level).

**Characteristic**	**Category**	**n (%)**	**Mean (SD)**
Age (years)			10.1 (1.1); n = 1126
MVPA (min/day)			100.1 (58.0); n = 1127
Screen time (min/day)			146.8 (131.8); n = 1126
BMI z-score			0.5 (1.2); n = 1126
KIDSCREEN (score)			41.2 (5.6); n = 1127
Sex	Male	579 (51.4%)	
	Female	548 (48.6%)	
	1	275 (24.4%)	
Wave	2	467 (41.4%)	
	3	385 (34.2%)	
	Private	269 (23.9%)	
School ownership	Charter	434 (38.5%)	
	Public	424 (37.6%)	
	G1	54 (4.8%)	
	G2	181 (16.1%)	
SES (G1–G4 + Missing)	G3	278 (24.7%)	
	G4	486 (43.1%)	
	Missing	128 (11.4%)	
Vegetables ≥ 2/day	Yes	458 (40.6%)	
Fish ≥ 2–3/week	Yes	807 (71.6%)	
Low HRQoL (KIDSCREEN < 40)	Yes	357 (31.7%)	

Notes. N observations: 1127; N children: 771; mean cluster size: 1.46 (median 1; range 1–3); repeated measures: 1 wave 60.6%, 2 waves 32.7%, 3 waves 6.7%. Abbreviations: MVPA, moderate-to-vigorous physical activity; SES, socioeconomic status. Exposures: vegetables ≥ 2/day and fish ≥ 2–3/week. Percentages are computed over observations. Counts and percentages for exposures are observation-level; children may contribute up to three observations.

## Data Availability

The data presented in this study are available on request from the corresponding author. The data are not publicly available due to privacy and ethical reasons.

## Supplementary Materials

The following supporting information can be downloaded at https://www.mdpi.com/article/10.3390/children13010056/s1, Figure S1: Overall adjusted probability of low HRQoL (KIDSCREEN < 40) across four fish × vegetable exposure combinations; Figure S2: Adjusted probability of low HRQoL (KIDSCREEN < 40) by socioeconomic status group and fish × vegetable exposure combinations; Figure S3: Forest plot from the hybrid within–between GEE (Mundlak) model for low HRQoL (KIDSCREEN < 40); Figure S4: Forest plot from sensitivity analysis including a KIDMED score excluding the fish and vegetable items (KIDMED_wo_FV); Figure S5: Forest plot from sensitivity analysis using tertiles of the total KIDMED score; Table S1: Marginal predicted probabilities of low HRQoL by sex and fish × vegetable exposure combinations; Table S2: Marginal predicted probabilities of low HRQoL by socioeconomic status group and fish × vegetable exposure combinations; Table S3: Marginal predicted probabilities of low HRQoL by measurement wave and fish × vegetable exposure combinations; Table S4: Marginal predicted probabilities of low HRQoL by school ownership and fish × vegetable exposure combinations. Table S5: Sensitivity of the primary GEE model to the working correlation structure. Table S6: Missing data in key variables and analytic sample size. Table S7: Sensitivity analysis excluding observations with missing SES.

## Abbreviations

The following abbreviations are used in this manuscript:

ASOMAD	Physical Activity, Sedentarism and Obesity in Madrid Study
BMI	Body mass index
CI	Confidence interval
EPA	Eicosapentaenoic acid
DHA	Docosahexaenoic acid
FFQ	Food-frequency questionnaire
GEE	Generalised estimating equations
HRQoL	Health-related quality of life
KIDMED	Mediterranean Diet Quality Index in children and adolescents
KIDMED_wo_FV	KIDMED score excluding the fish and vegetable items
LC n-3 PUFA	Long-chain n-3 polyunsaturated fatty acids
MVPA	Moderate-to-vigorous physical activity
OR	Odds ratio
PAU-7S	Physical Activity Unit 7-item Screener
SD	Standard deviation
SES	Socioeconomic status
SSBQ	Screen-based Sedentary Behaviour Questionnaire
STROBE	Strengthening the Reporting of Observational Studies in Epidemiology
WHtR	Waist-to-height ratio
