# Supplementary material for: Do Single Food Habits Matter? Fish and Vegetables Intake and Risk of Low HRQoL in Schoolchildren (ASOMAD Study)
Source: Children (Basel). 2025 Dec 30;13(1):56. doi: 10.3390/children13010056 (PMC12840302; doi:10.3390/children13010056)
Supplement: Supplementary file 1 [file children-13-00056-s001.zip › Children/Captions_Supplementary_Figures_S1_S5.pdf]

Supplementary Figure S1. Adjusted probability of low emotional well-being (KIDSCREEN < 40) across four fish × vegetable exposure combinations.

Note. Bars represent marginal predicted probabilities of reporting low emotional well-being (KIDSCREEN < 40) for children in each of the four exposure combinations: 00 = vegetables <2 servings/day and fish <2–3 times/week (None); 10 = vegetables ≥2 servings/day only (Vegetables only); 01 = fish ≥2–3 times/week only (Fish only); 11 = both behaviours (Both). Estimates are derived from the main GEE model adjusted for age, sex, measurement wave, socioeconomic status and school ownership. Error bars indicate 95% confidence intervals. KIDSCREEN = KIDSCREEN-10 health-related quality of life index; GEE = generalized estimating equations.

Supplementary Figure S2. Adjusted probability of low emotional well-being (KIDSCREEN < 40) by socioeconomic status group and fish × vegetable exposure combinations.

Note. Bars represent marginal predicted probabilities of reporting low emotional well-being (KIDSCREEN < 40) for each combination of fish and vegetable intake (00, 10, 01, 11 as defined in Supplementary Figure S1), stratified by socioeconomic status (SES) groups G1–G4 from the multidimensional SES index. Estimates are derived from the main GEE model and are adjusted for age, sex, measurement wave and school ownership. Error bars indicate 95% confidence intervals. SES = socioeconomic status; KIDSCREEN = KIDSCREEN-10 health-related quality of life index; GEE = generalized estimating equations.

Supplementary Figure S3. Forest plot from the hybrid within–between GEE (Mundlak) model for low emotional well-being (KIDSCREEN < 40).

Note. Odds ratios (OR) and 95% confidence intervals are shown for fish and vegetable intake (between-child means and within-child deviations) and all covariates in the hybrid within–between GEE (Mundlak) model with low emotional well-being (KIDSCREEN < 40) as the dependent variable. The vertical dashed line indicates the null value (OR = 1.0). KIDSCREEN = KIDSCREEN-10 health-related quality of life index; OR = odds ratio; GEE = generalized estimating equations.

Supplementary Figure S4. Forest plot from sensitivity analysis including a KIDMED score excluding the fish and vegetable items (KIDMED_wo_FV).

Note. Odds ratios (OR) and 95% confidence intervals are shown for vegetables ≥2 servings/day, fish ≥2–3 times/week, their interaction term and covariates in a GEE model additionally adjusted for KIDMED_wo_FV (KIDMED score excluding the fish and vegetable items), entered as a continuous variable. The vertical dashed line indicates the null value (OR = 1.0). KIDMED_wo_FV = KIDMED score excluding fish and vegetable items; KIDMED = Mediterranean Diet Quality Index in children and adolescents; OR = odds ratio; GEE = generalized estimating equations.

Supplementary Figure S5. Forest plot from sensitivity analysis using tertiles of the total KIDMED score.

Note. Odds ratios (OR) and 95% confidence intervals are shown for KIDMED tertiles (medium and high vs low) and covariates in a GEE model for low emotional well-being (KIDSCREEN < 40). The vertical dashed line indicates the null value (OR = 1.0). KIDSCREEN = KIDSCREEN-10 health-related quality of life index; KIDMED = Mediterranean Diet Quality Index in children and adolescents; OR = odds ratio; GEE = generalized estimating equations.
