# Supplementary material for: Do Single Food Habits Matter? Fish and Vegetables Intake and Risk of Low HRQoL in Schoolchildren (ASOMAD Study)
Source: Children (Basel). 2025 Dec 30;13(1):56. doi: 10.3390/children13010056 (PMC12840302; doi:10.3390/children13010056)
Supplement: Supplementary file 1 [file children-13-00056-s001.zip › Children/Supplementary_Table_S1.pdf]

**Supplementary Table S1**. Marginal predicted probabilities of low emotional well-being by sex and fish × vegetable scenarios

| Stratum (sex) | Scenario | Risk %, 95% CI | Δ p.p. vs 00, 95% CI |
|---|---|---|---|
| Female | 00 None | 44.0 (36.2–51.7) | 0.0 (0.0–0.0) |
| | 10 Vegetables only | 28.7 (20.1–37.3) | -15.3 (-25.8–-4.7) |
| | 01 Fish only | 32.9 (27.7–38.1) | -11.1 (-19.2–-3.0) |
| | 11 Both | 37.5 (31.6–43.4) | -6.5 (-15.2–2.3) |
| Male | 00 None | 36.5 (29.4–43.5) | 0.0 (0.0–0.0) |
| | 10 Vegetables only | 23.0 (15.6–30.3) | -13.5 (-22.8–-4.2) |
| | 01 Fish only | 26.6 (21.9–31.3) | -9.9 (-17.2–-2.5) |
| | 11 Both | 30.6 (24.9–36.3) | -5.8 (-13.8–2.1) |

Note. Δ p.p. = absolute difference versus scenario 00, in percentage points; CI = confidence interval.
