# Supplementary material for: Do Single Food Habits Matter? Fish and Vegetables Intake and Risk of Low HRQoL in Schoolchildren (ASOMAD Study)
Source: Children (Basel). 2025 Dec 30;13(1):56. doi: 10.3390/children13010056 (PMC12840302; doi:10.3390/children13010056)
Supplement: Supplementary file 1 [file children-13-00056-s001.zip › Children/Supplementary_Table_S2.pdf]

Supplementary Table S2. Marginal predicted probabilities of low emotional well-being by socioeconomic status group and fish × vegetable scenarios

Stratum (SES group)	Scenario	Risk %, 95% CI	Δ p.p. vs 00, 95% CI
G1	00 None	55.0 (40.0–70.0)	0.0 (0.0–0.0)
	10 Vegetables only	38.4 (22.5–54.3)	-16.6 (-28.1–-5.0)
	01 Fish only	43.2 (28.4–58.0)	-11.8 (-20.2–-3.4)
	11 Both	48.2 (33.2–63.2)	-6.7 (-15.8–2.3)
G2	00 None	34.8 (26.0–43.5)	0.0 (0.0–0.0)
	10 Vegetables only	21.4 (13.4–29.3)	-13.4 (-22.8–-4.1)
	01 Fish only	24.9 (18.3–31.5)	-9.9 (-17.2–-2.5)
	11 Both	28.9 (21.4–36.4)	-5.9 (-13.8–2.1)
G3	00 None	46.0 (37.1–54.9)	0.0 (0.0–0.0)
	10 Vegetables only	30.2 (20.4–40.1)	-15.7 (-26.6–-4.9)
	01 Fish only	34.6 (28.3–40.9)	-11.4 (-19.7–-3.1)
	11 Both	39.3 (31.9–46.8)	-6.6 (-15.5–2.3)
G4	00 None	33.3 (25.7–41.0)	0.0 (0.0–0.0)
	10 Vegetables only	20.3 (13.0–27.5)	-13.1 (-22.1–-4.1)
	01 Fish only	23.7 (18.8–28.6)	-9.7 (-16.9–-2.4)
	11 Both	27.6 (22.0–33.2)	-5.7 (-13.6–2.1)

Note. Δ p.p. = absolute difference versus scenario 00, in percentage points; CI = confidence interval; SES = socioeconomic status.
