# Supplementary material for: Do Single Food Habits Matter? Fish and Vegetables Intake and Risk of Low HRQoL in Schoolchildren (ASOMAD Study)
Source: Children (Basel). 2025 Dec 30;13(1):56. doi: 10.3390/children13010056 (PMC12840302; doi:10.3390/children13010056)
Supplement: Supplementary file 1 [file children-13-00056-s001.zip › Children/Supplementary_Table_S3.pdf]

**Supplementary Table S3.** Marginal predicted probabilities of low emotional well-being by measurement wave and fish × vegetable scenarios

| Stratum (wave) | Scenario | Risk %, 95% CI | Δ p.p. vs 00, 95% CI |
|---|---|---|---|
| 1 | 00 None | 43.0 (35.0–51.0) | 0.0 (0.0–0.0) |
| | 10 Vegetables only | 28.1 (19.3–36.9) | -14.9 (-25.1–-4.6) |
| | 01 Fish only | 32.2 (25.7–38.6) | -10.8 (-18.6–-2.9) |
| | 11 Both | 36.7 (29.9–43.4) | -6.3 (-14.8–2.2) |
| 2 | 00 None | 40.2 (32.8–47.5) | 0.0 (0.0–0.0) |
| | 10 Vegetables only | 25.9 (18.1–33.6) | -14.3 (-24.2–-4.4) |
| | 01 Fish only | 29.7 (25.1–34.4) | -10.4 (-18.1–-2.7) |
| | 11 Both | 34.0 (28.0–40.1) | -6.1 (-14.4–2.1) |
| 3 | 00 None | 38.0 (30.1–45.9) | 0.0 (0.0–0.0) |
| | 10 Vegetables only | 24.0 (15.9–32.1) | -14.0 (-23.6–-4.4) |
| | 01 Fish only | 27.7 (22.6–32.9) | -10.2 (-17.9–-2.6) |
| | 11 Both | 31.9 (26.2–37.7) | -6.0 (-14.3–2.2) |

Note. Δ p.p. = absolute difference versus scenario 00, in percentage points; CI = confidence interval.
