# Supplementary material for: Do Single Food Habits Matter? Fish and Vegetables Intake and Risk of Low HRQoL in Schoolchildren (ASOMAD Study)
Source: Children (Basel). 2025 Dec 30;13(1):56. doi: 10.3390/children13010056 (PMC12840302; doi:10.3390/children13010056)
Supplement: Supplementary file 1 [file children-13-00056-s001.zip › Children/Supplementary_Table_S4.pdf]

Supplementary Table S4. Marginal predicted probabilities of low emotional well-being by school ownership and fish × vegetable scenarios.

Stratum (school ownership)	Scenario	Risk %, 95% CI	Δ p.p. vs 00, 95% CI
Private	00 None	32.5 (23.6–41.5)	0.0 (0.0–0.0)
	10 Vegetables only	19.7 (11.7–27.7)	-12.8 (-21.7–-3.9)
	01 Fish only	23.1 (16.9–29.3)	-9.5 (-16.7–-2.3)
	11 Both	26.9 (20.0–33.8)	-5.6 (-13.4–2.1)
Charter	00 None	43.1 (35.2–50.9)	0.0 (0.0–0.0)
	10 Vegetables only	28.2 (19.8–36.7)	-14.8 (-25.1–-4.6)
	01 Fish only	32.3 (26.9–37.7)	-10.7 (-18.6–-2.9)
	11 Both	36.8 (30.2–43.4)	-6.3 (-14.8–2.2)
Public	00 None	41.9 (34.2–49.6)	0.0 (0.0–0.0)
	10 Vegetables only	27.1 (18.6–35.6)	-14.8 (-25.1–-4.6)
	01 Fish only	31.1 (25.6–36.7)	-10.8 (-18.7–-2.9)
	11 Both	35.6 (29.3–41.8)	-6.3 (-14.9–2.2)

Note. Δ p.p. = absolute difference versus scenario 00, in percentage points; CI = confidence interval.
