# Supplementary figures and images for: Do Single Food Habits Matter? Fish and Vegetables Intake and Risk of Low HRQoL in Schoolchildren (ASOMAD Study)
Source: Children (Basel). 2025 Dec 30;13(1):56. doi: 10.3390/children13010056 (PMC12840302; doi:10.3390/children13010056)

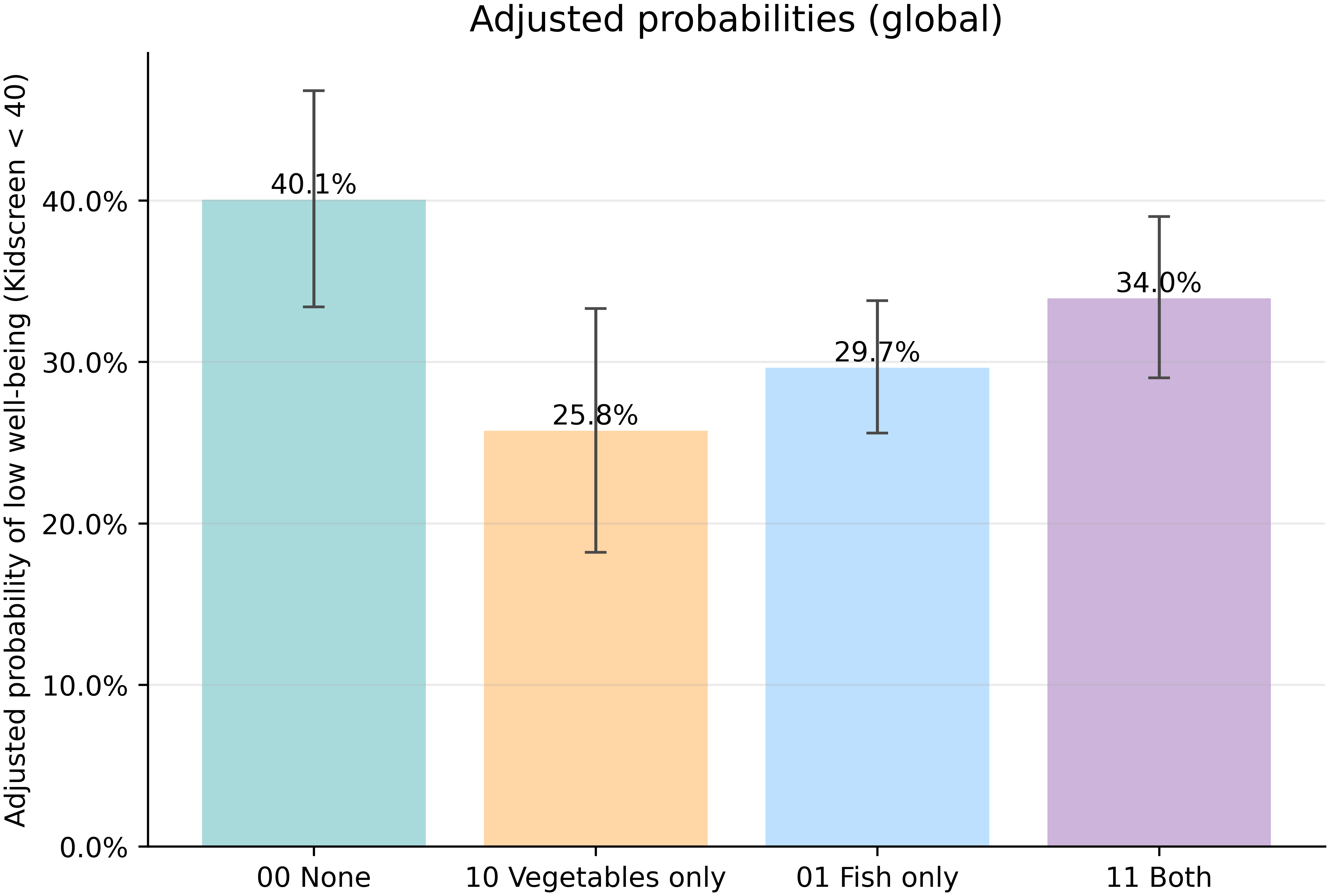

Supplement: Supplementary file 1 [file children-13-00056-s001.zip › Children/Supplementary Figure S1.png]

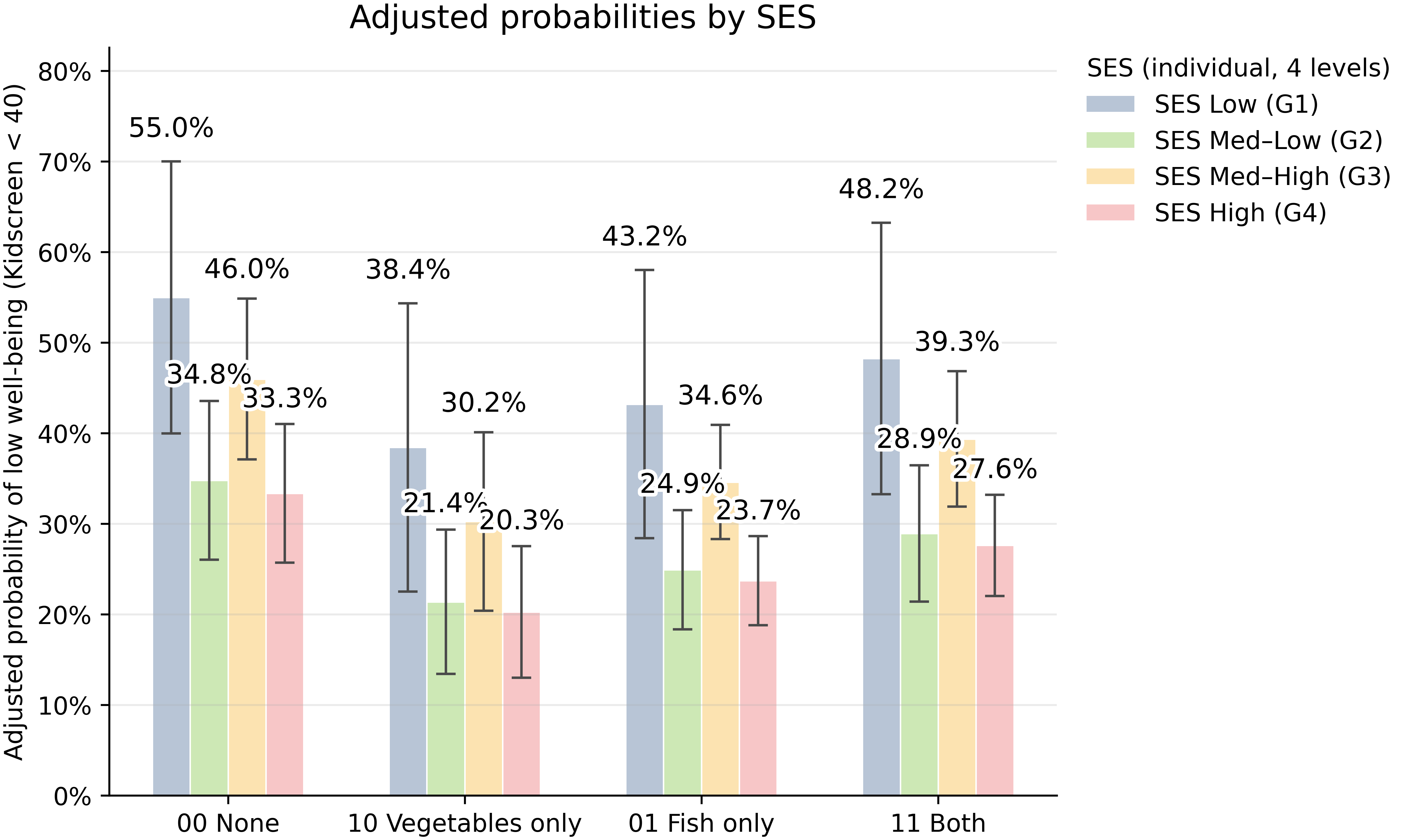

Supplement: Supplementary file 1 [file children-13-00056-s001.zip › Children/Supplementary Figure S2.png]

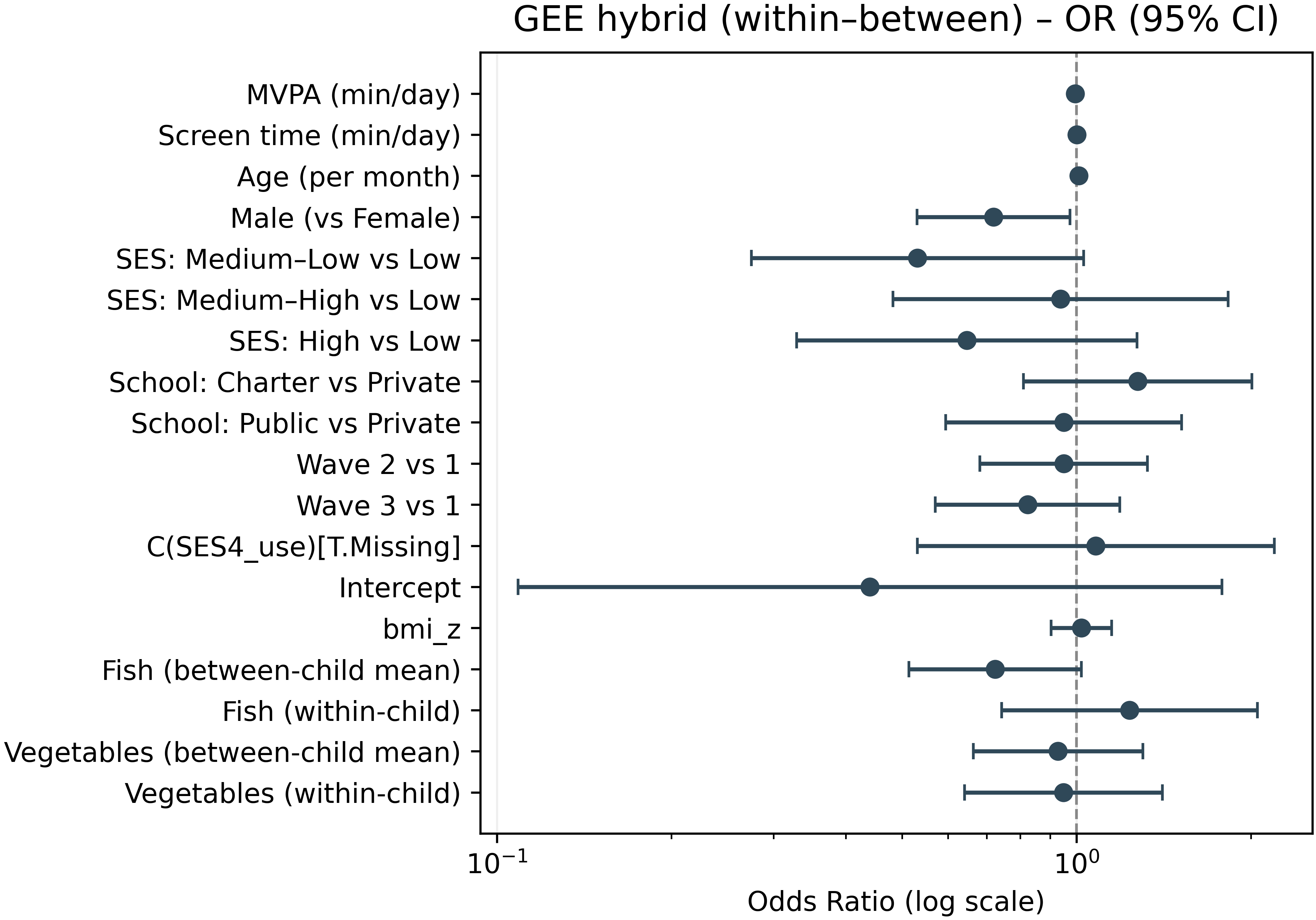

Supplement: Supplementary file 1 [file children-13-00056-s001.zip › Children/Supplementary Figure S3.png]

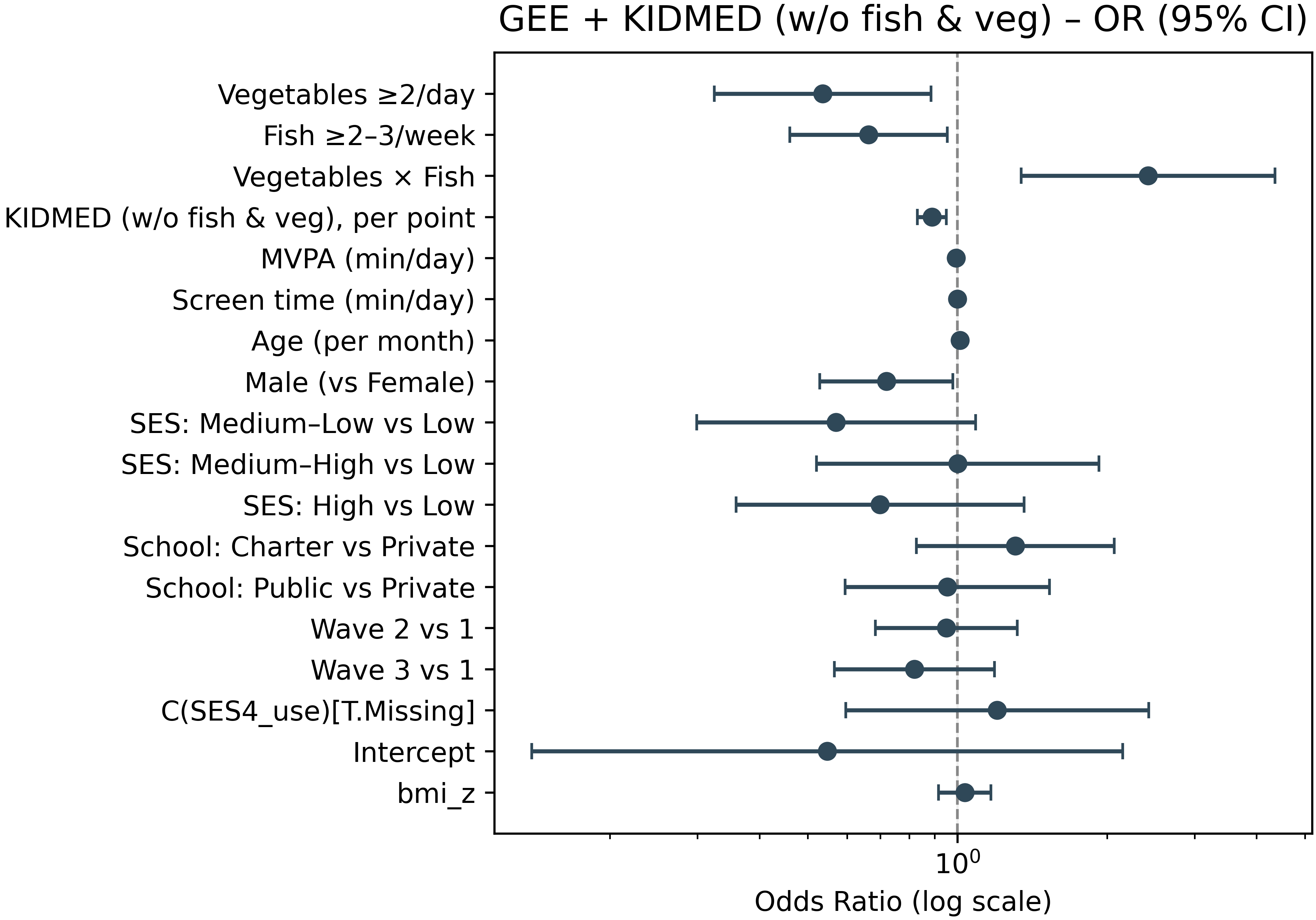

Supplement: Supplementary file 1 [file children-13-00056-s001.zip › Children/Supplementary Figure S4.png]

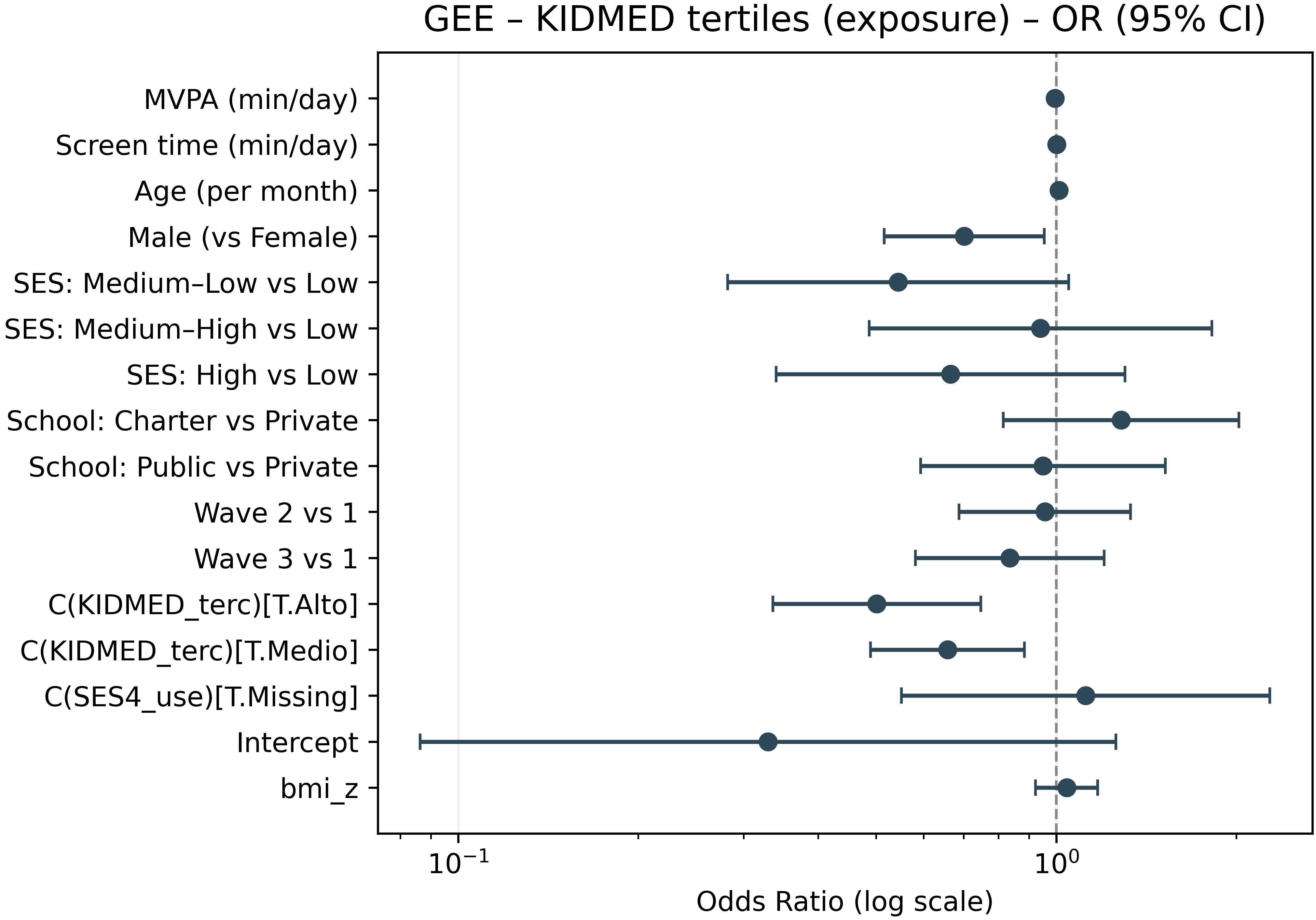

Supplement: Supplementary file 1 [file children-13-00056-s001.zip › Children/Supplementary Figure S5.png]
